# Optimised deep neural network model to predict asthma exacerbation based on personalised weather triggers

**DOI:** 10.12688/f1000research.73026.1

**Published:** 2021-09-10

**Authors:** Radiah Haque, Sin-Ban Ho, Ian Chai, Adina Abdullah

**Affiliations:** 1 Faculty of Computing and Informatics, Multimedia University, Cyberjaya, 63100, Malaysia; 2 Faculty of Medicine, University of Malaya, Kuala Lumpur, 50603, Malaysia

**Keywords:** Machine learning, deep neural network, personalisation, asthma self-management

## Abstract

**Background** – Recently, there have been attempts to develop mHealth applications for asthma self-management. However, there is a lack of applications that can offer accurate predictions of asthma exacerbation using the weather triggers and demographic characteristics to give tailored response to users. This paper proposes an optimised Deep Neural Network Regression (DNNR) model to predict asthma exacerbation based on personalised weather triggers.

**Methods** – With the aim of integrating weather, demography, and asthma tracking, an mHealth application was developed where users conduct the Asthma Control Test (ACT) to identify the chances of their asthma exacerbation. The asthma dataset consists of panel data from 10 users that includes 1010 ACT scores as the target output. Moreover, the dataset contains 10 input features which include five weather features (temperature, humidity, air-pressure, UV-index, wind-speed) and five demography features (age, gender, outdoor-job, outdoor-activities, location).

**Results** – Using the DNNR model on the asthma dataset, a score of 0.83 was achieved with Mean Absolute Error (MAE)=1.44 and Mean Squared Error (MSE)=3.62. It was recognised that, for effective asthma self-management, the prediction errors must be in the acceptable loss range (error<0.5). Therefore, an optimisation process was proposed to reduce the error rates and increase the accuracy by applying standardisation and fragmented-grid-search. Consequently, the optimised-DNNR model (with 2 hidden-layers and 50 hidden-nodes) using the Adam optimiser achieved a 94% accuracy with MAE=0.20 and MSE=0.09.

**Conclusions** – This study is the first of its kind that recognises the potentials of DNNR to identify the correlation patterns among asthma, weather, and demographic variables. The optimised-DNNR model provides predictions with a significantly higher accuracy rate than the existing predictive models and using less computing time. Thus, the optimisation process is useful to build an enhanced model that can be integrated into the asthma self-management for mHealth application.

## Introduction

Asthma is a chronic lung disease that affects people of all age groups around the world. ^1^ Asthma exacerbation causes asthma attacks, and the frequency of asthma attacks depends on the exposure to asthma triggers. ^2^ Weather is a common triggering factor of asthma exacerbation. ^3^ Studies show that weather triggers, such as temperature, humidity, air pressure, and wind, cause asthma attacks. ^4– 6^ Weather impact is specific to individual asthmatic patients due to their lung performance, which varies among patients. This depends on their demographic characteristics, such as age and gender. ^7^ Geographical location is also a factor because the association between weather triggers and asthma is inconsistent in different climate regions. ^4^

Although asthma cannot be cured, avoiding exposure to weather triggers through asthma self-management can minimise the risk of asthma exacerbation. ^8^ Recently, there have been attempts to develop mHealth applications to assist asthma self-management. However, until now, no application for effective asthma self-management exists that has been widely adopted by users or integrated into primary asthma care records. ^2^ This is because there is a lack of solutions that can offer accurate predictions of asthma exacerbation based on personalised weather triggers and provide tailored feedback to users.

Deep Neural Network (DNN) is a type of neural network algorithm with multiple hidden layers and several nodes. ^9^ In recent years, DNN has been significantly utilised in the health informatics research domain for forecasting and pattern recognition. ^10– 13^ This is because DNN models tend to learn more effectively and have better performance in providing accurate predictions (especially through optimisation) than traditional Machine Learning (ML) algorithms. ^14^ Nevertheless, the application of ML and DNN in weather-based healthcare is still in its infancy. In fact, to the best of the authors’ knowledge, none of the existing research has applied DNN to predict asthma exacerbation based on demography and weather. Therefore, the main contribution of the work in this paper is to apply DNN and propose an optimisation process to predict asthma exacerbation based on personalised weather triggers with low error and high accuracy. The findings will be helpful for developing mHealth solutions with personalisation for effective asthma self-management.

## Methods

### Data collection

With the aim of integrating weather, demography, and asthma tracking, an mHealth application, namely Weather Asthma (WEA), was developed for this study. ^15^ The WEA is an android-based application that collects user demography and monitors daily weather forecasts in individual users’ location to identify the potential weather triggers. Consequently, both demography and weather data are selected as input features in the asthma dataset.

The WEA application also allows users to conduct the Asthma Control Test (ACT). ^16^ The ACT is a self-administered survey which is considered the standard assessment for monitoring chronic asthma and recommended by the Global Initiative for Asthma. ^17^ The ACT score is selected as the target output for prediction because it helps identify the severity and chances of asthma exacerbation. ^16^

Data was collected through the WEA application from ten participants with asthma over a period of one-year. Participants conducted ACTs by regularly answering five multiple-choice questions, which include four asthma symptom-related questions and one asthma self-evaluate question. Each question is scored between 1-5. Once the ACT was submitted, a timestamp was formed with the participant’s demography and the weather information of that day and time at their location. This timestamp, along with the total ACT score, are stored in the database, as seen in Table 1. All participants consented to the data collection and the ethical approval was obtained from the Multimedia University Research Ethics Committee (EA1532021).

**Table 1.** Data collection example.

Data name	Example
User ID	5QBEe959GPOxv3rDNYXZX
Timestamp	1607926290609
ACT score	20
Age	Above 50
Gender	Male
Outdoor job	Occasionally
Outdoor activites	Extremely likely
Smoking habit	No
Temperature	29.8°C
Humidity	70%
Air pressure	1009.0 hectopascals
UV index	Low (i.e. 0 to 2)
Wind speed	2.1 meters per second

### Data pre-processing

**Figure 1.**
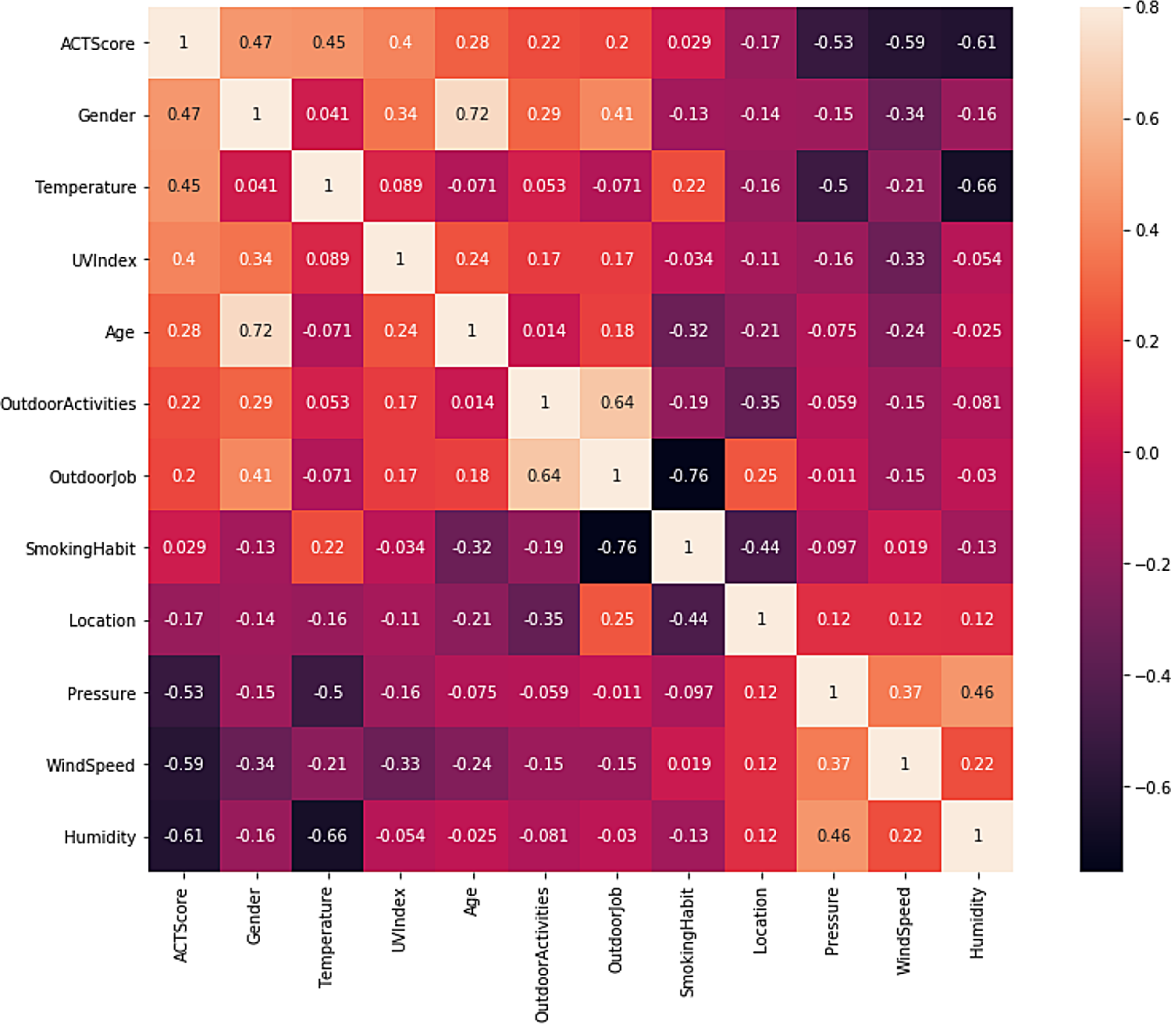
Heatmap visualisation.

The first step of data pre-processing is identifying the missing data in the dataset through a heatmap, illustrated in Figure 1, which visualises the locations of missing values. Fortunately, the selected dataset does not contain any missing or NaN values.

**Figure 2.**
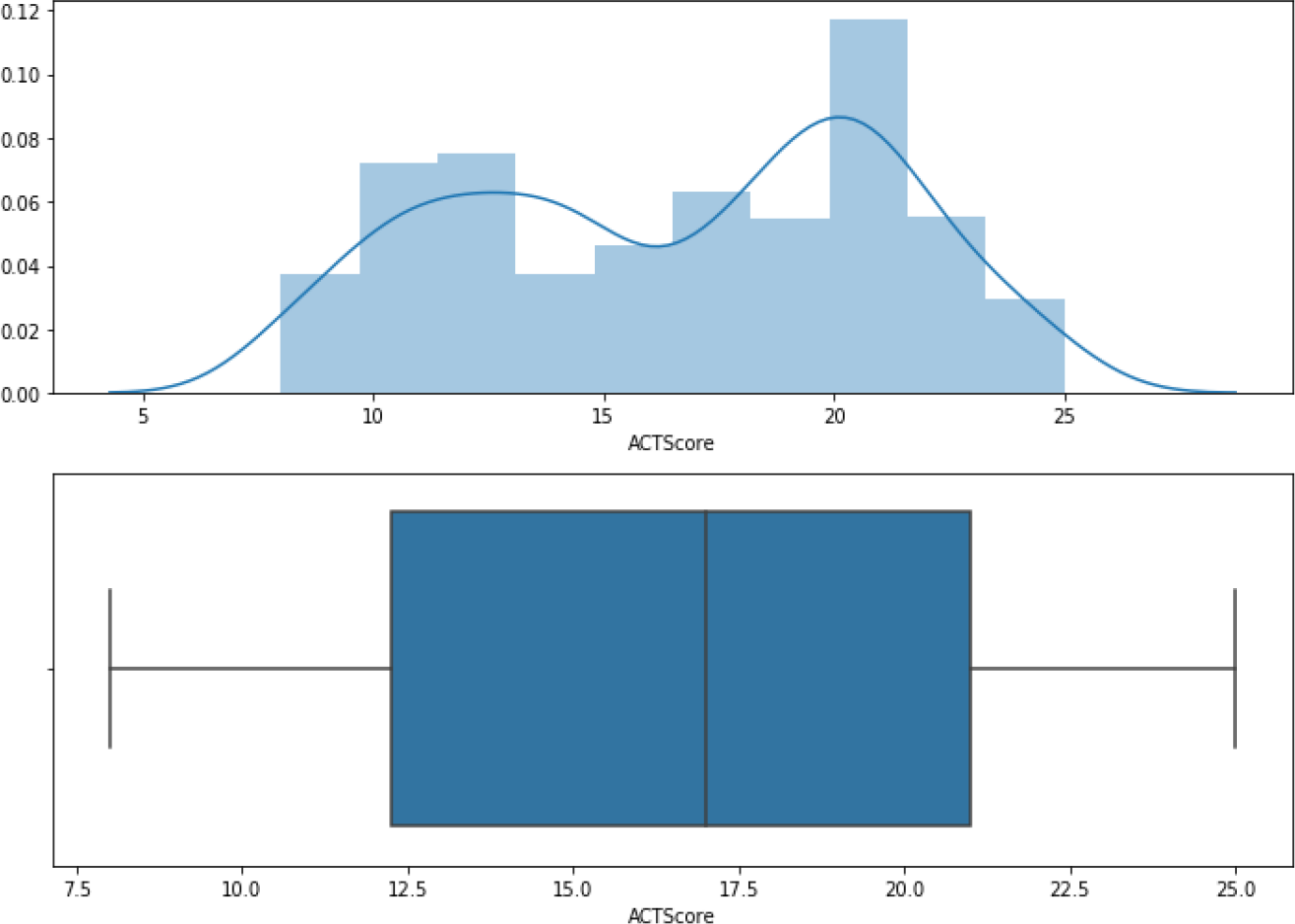
ACT distribution.

**Figure 3.**
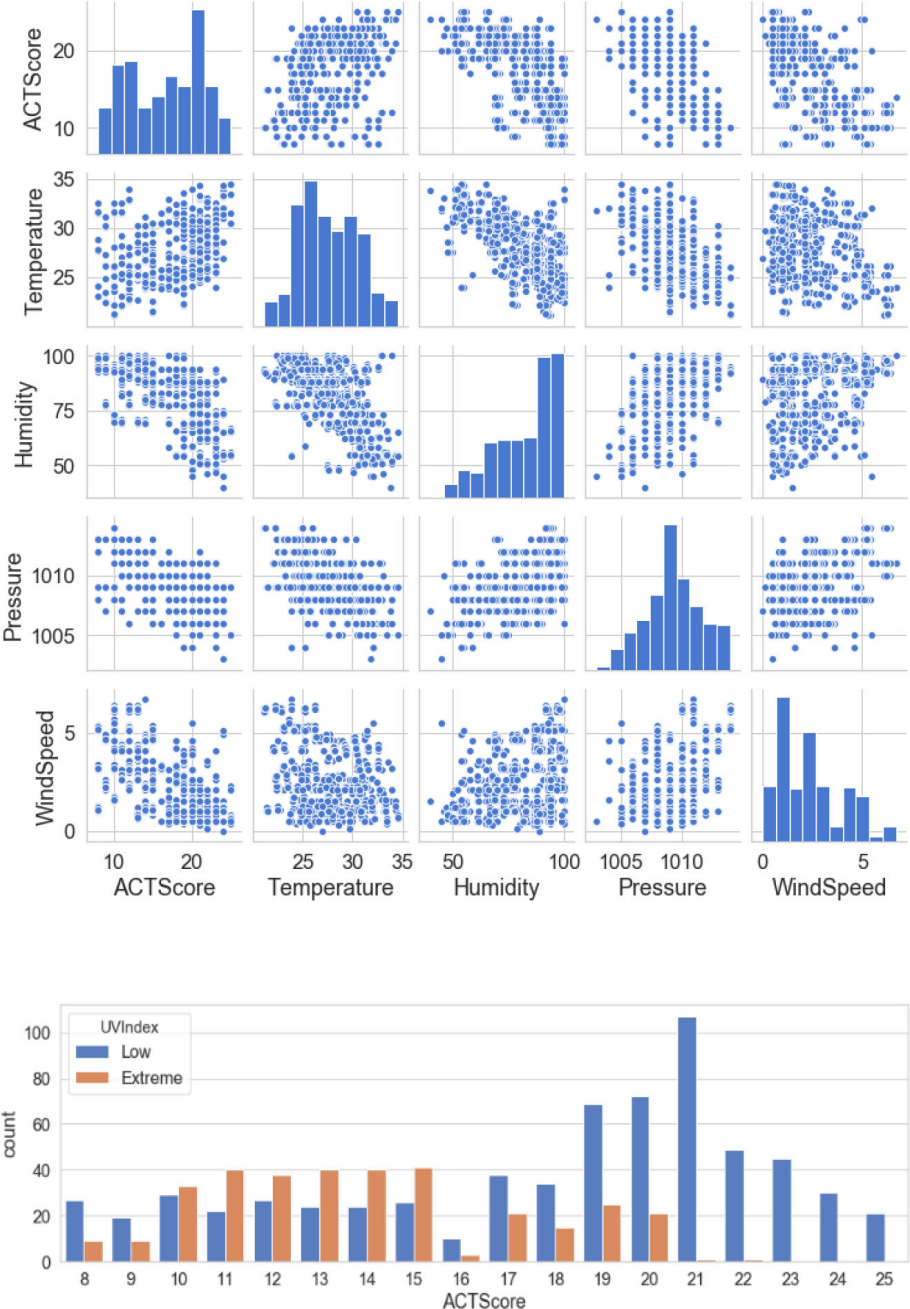
Weather visualisation.

The second step is dropping irrelevant features including “User ID” and “Timestamp”. “Smoking habit” is also dropped because its correlation coefficient value with the target variable “ACT score” in the heatmap is close to zero. Table 2 represents the final dataset, which consists of 1010 records with ten input features and one output variable. Figure 2 shows the ACT scores’ distribution, which ranges from 12 to 21. Figure 3 illustrates a scatterplot and countplot for weather features, where a strong correlation can be observed between the weather features and “ACT score”.

**Table 2.** Input/output variables.

Name	Type	Description
Age	Categorical	Input demography
Gender	Categorical	Input demography
Location	Categorical	Input demography
Outdoor activities	Categorical	Input demography
Outdoor job	Categorical	Input demography
Temperature	Numerical	Input weather
Humidity	Numerical	Input weather
Air pressure	Numerical	Input weather
UV index	Categorical	Input weather
Wind speed	Numerical	Input weather
ACT score	Numerical	Target output

The third step is converting the categorical variables in the dataset to numeric representations using the label encoder. The fourth step is splitting the dataset into training (707 samples) and testing (303 samples) datasets.

### Regression with DNN

DNN can be modelled with various ML techniques, such as regression and classification. ^18^ Regression is responsible for modelling and characterising the relationship between the input features and the target output. Regression is applied to predict numerical values. ^19^ Hence, regression is used in this study to predict the ACT score, which is a numerical value.

**Figure 4.**
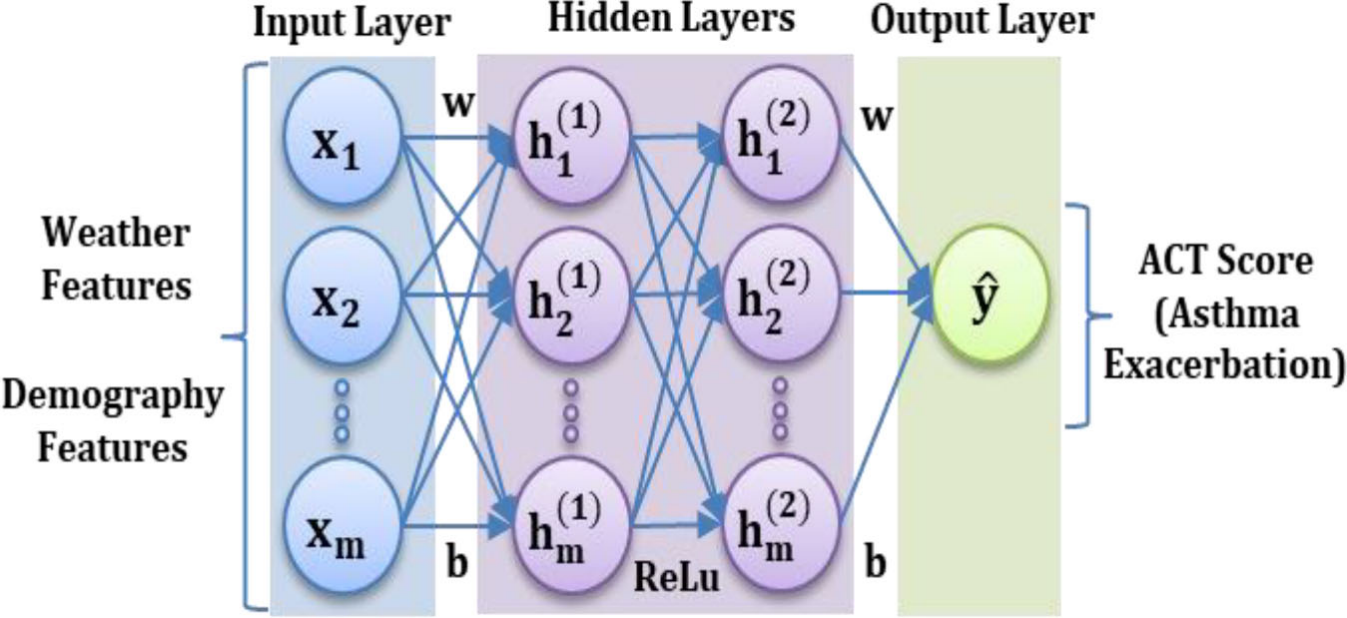
DNNR architecture.

Consequently, a DNN Regression (DNNR) model is applied on the dataset. In DNNR, the hidden layers are located between the input layer and the output layer, as seen in Figure 4. The hidden layers apply weights to input values and direct them via an activation function for the output values. ^20^ The activation function assists in deriving distinguishing features that are required for the prediction. ^10^ This is particularly helpful to model the asthma dataset which contains multiple types of input features. The Rectified Linear Unit (ReLU) activation function is used because it provides nonlinear transformations for deep modelling. ^9^

The following are the main equations used for prediction using the DNNR model:

$$x=\left[x_{1},x_{2},...,x_{m}\right]w=\left[w_{1},w_{2},...,w_{m}\right]f\left(e\right)=\{\begin{array}{lll} 0 & for & e\;<\;0\\ e & for & e\;\;\geq \;\;0 \end{array}e=\sum_{j=1}^{m}xw+b\hat{y}=f\left(e\right)$$

where *x* is the input features, $\hat{y}$ is the predicted values, *w* is the input weights, *b* is the bias (a constant number used for adjustment), *e* is the internal elements in the hidden layers, *f*( *e*) is the activation function, *m* is the number of input features, and *j* is a constant number between [0, *m*].

### Evaluation metrics

Evaluating the DNNR model is essential to determine its prediction error and accuracy, which can be achieved through Mean Absolute Error (MAE), Mean Squared Error (MSE), and Explained Variance Score (EVS). The MAE sums up the absolute difference between the actual and the predicted values. The MSE sums up the squared differences between the actual and the predicted values. The EVS computes the variance score which determines the accuracy of nonlinear regression models. ^9^ The following equations calculate the MAE, MSE and EVS of the DNNR model:

$$\mathrm{MAE}=\frac{1}{n}\sum_{i-1}^{n}\left|{\hat{y}}_{i}-y_{i}\right|\mathrm{MSE}=\frac{1}{n}\sum_{i-1}^{n}{\left({\hat{y}}_{i}-y_{i}\right)}^{2}\mathrm{EVS}=1-\frac{v\left(\hat{y}-y\right)}{v\left(y\right)}$$

where *y* is the actual output values, $\hat{y}$ is the predicted values, *n* is the number of records in the dataset, *v* is the biased variance, and *i* is a constant number between [0, *n*].

### Optimisation methods

Optimising the DNNR model is crucial for prediction with low error, high accuracy, and less computing time. This can be achieved by applying essential optimisation methods which include data scaling and parameter tuning. For data scaling, standardisation is used because it is beneficial for enhancing the performance of the DNNR model and its optimisation. ^9^ This happens by rescaling the input and the output values using the following equations:

$$\begin{array}{l} s^{\prime }=\frac{s-\mu }{\sigma }\\ \mu =\frac{1}{n}\sum_{i-1}^{n}s_{i}\\ \sigma =\sqrt{\frac{1}{n}\sum_{i-1}^{n}{\left(s_{i}-\mu \right)}^{2}} \end{array}$$

where *s* is the input/output variables, *s*′ is the standardised input/output values, μ is the mean of the input/output values, σ is the standard deviation of the input/output values, *n* is the number of records in the dataset, and *i* is a constant number between [0, *n*].

**Figure 5.**
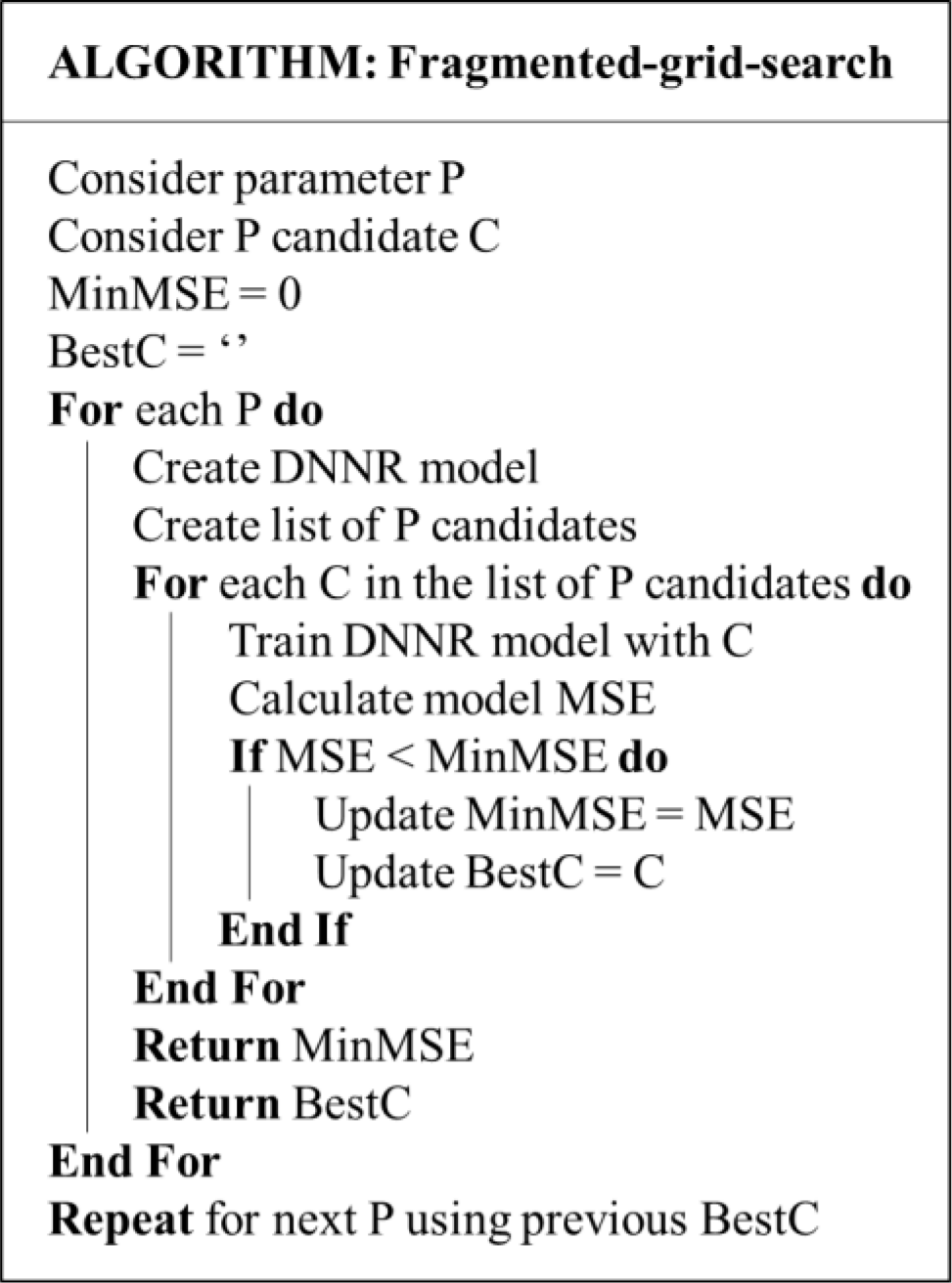
Optimisation algorithm.

**Figure 6.**
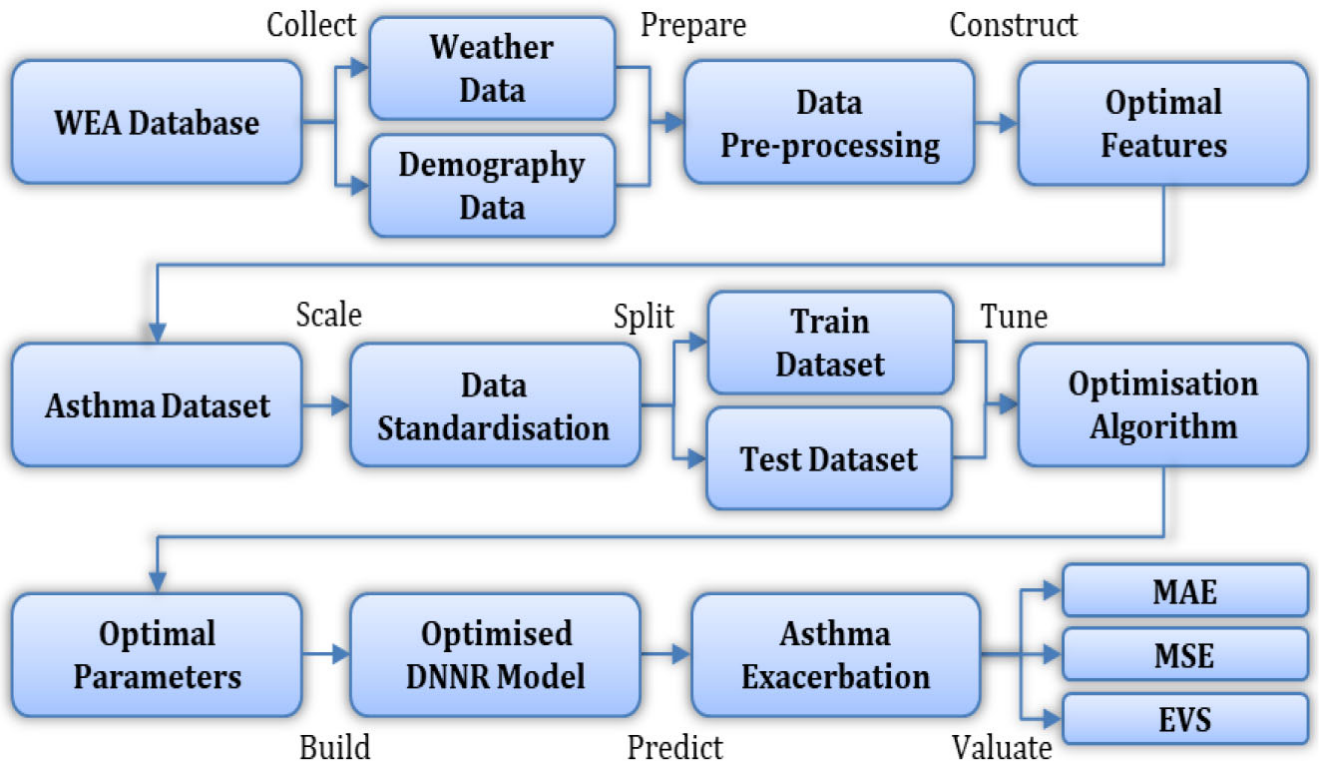
Optimisation process.

The DNNR parameters include hidden layers, nodes at each hidden layer, batch size, epochs, weight initialiser, loss function, and optimiser. Grid-search is an optimisation algorithm which automates the trial procedure of tuning these parameters and selecting their best values. ^21^ Nevertheless, tuning a large number of parameters and their search values using grid-search leads to excessive computational time and power. In this study, the fragmented-grid-search method is used where parameters are tuned independently in parallel, hence taking less computing time for optimisation. Figure 5 demonstrates the optimisation algorithm and Figure 6 illustrates the overall optimisation process.

## Results

### Regression results

**Figure 7.**
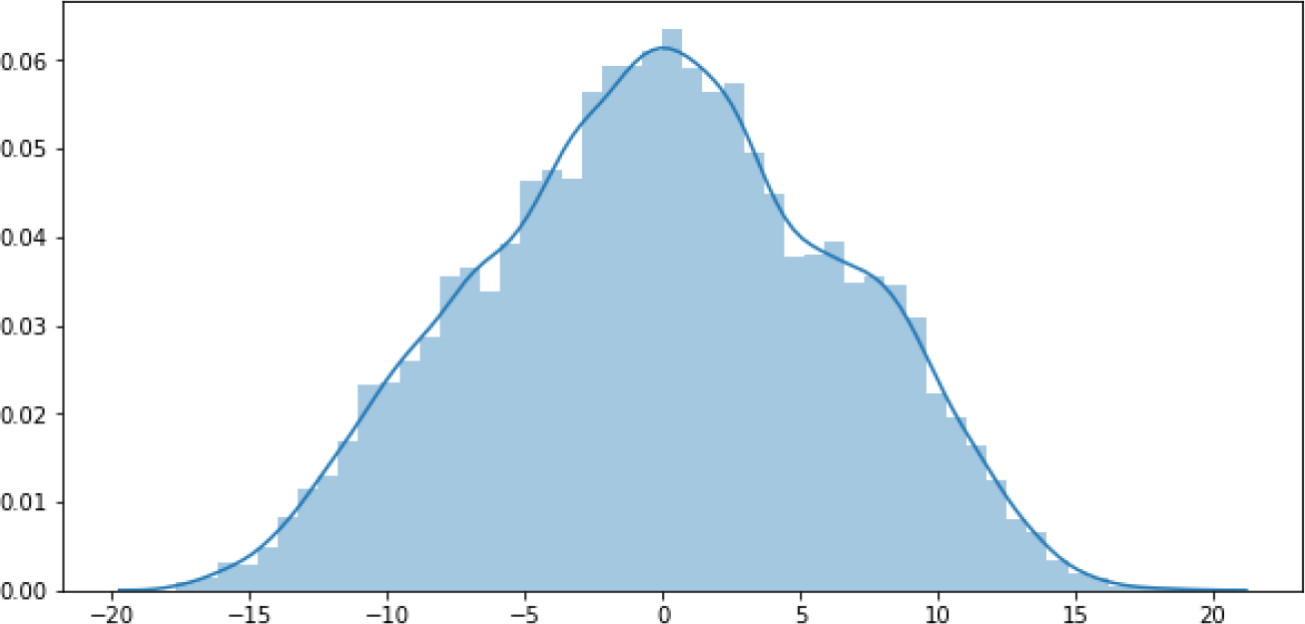
DNNR Residual.

Using the DNNR model on the dataset, a score of 0.83 is achieved with MAE = 1.44 and MSE = 3.62. Table 3 shows 5 predicted values against their actual values and Figure 7 contains the residual visualisation. It can be seen that the differences between the predicted and the actual values vary up to ±15. While this might seem an acceptable prediction error for some datasets, in the case of the asthma dataset, this amount of loss is unacceptable. This is because the range of the ACT score can only be from 5 to 25, where scores of 5 to 15 are categorised as “poorly-controlled asthma”, 16 to 19 as “not well-controlled asthma”, and 20 to 25 as “well-controlled asthma”. ^17^

In the last row of Table 3, with the actual value of 19 (not well-controlled), the predicted value is 20 (well-controlled), which gives a contradictory prediction result. This can be a serious problem while providing tailored feedback to asthmatic patients, resulting in an insufficiently effective asthma self-management solution. For an optimised model, the acceptable loss range needs to be less than ±0.5. For example, with the actual value of 19, the prediction value can be at most 19.4≃19 (with maximum +0.4 loss) or at least 18.6≃19 (with maximum −0.4 loss). Therefore, an optimised-DNNR model is built to reduce the prediction error and increase the overall accuracy.

**Table 3.** Actual vs. predicted values.

Actual values	Group	Predicted values	Group
12	Poorly-controlled	16	Not well-controlled
25	Well-controlled	19	Not well-controlled
17	Not well-controlled	22	Well-controlled
21	Well-controlled	18	Not well-controlled
19	Not well-controlled	20	Well-controlled

### Optimisation results

**Figure 8.**
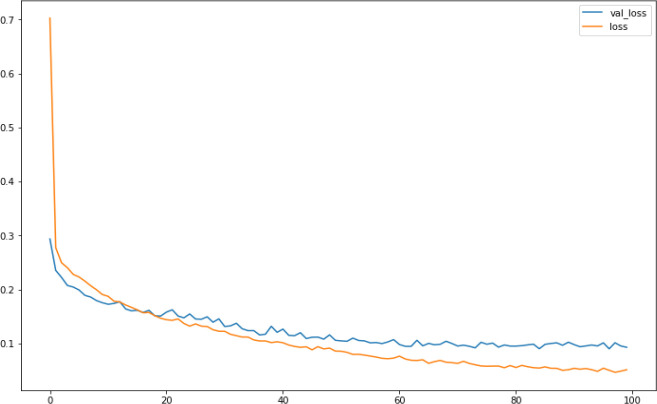
Loss function.

For the optimised-DNNR model, two hidden layers are used with 50 nodes at each hidden layer. Adaptive Moment Estimation (Adam) is used as the optimiser, which is helpful for optimising the learning and convergence rates during model training. ^13^ Table 4 summarises the optimum parameter values obtained using fragmented-grid-search and the total tuning time. Figure 8 shows the loss rate of the training and the testing datasets swiftly decreased using the ReLU activation function.

**Table 4.** Optimum parameters.

No. of hidden layers	2
No. of nodes	50
Batch size	10
No. of epochs	100
Loss function	MSE
Optimiser	Adam
Weight initialiser	Normal
Total tuning time	26 minutes

**Figure 9.**
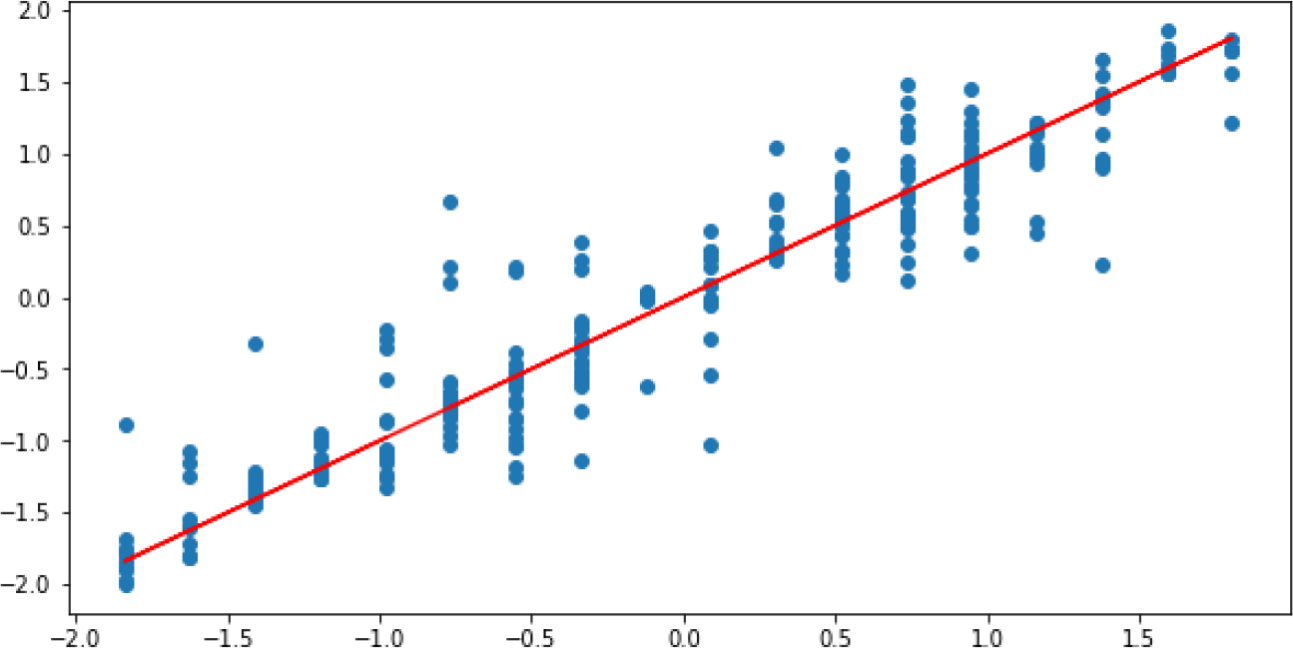
Optimised-DNNR prediction.

**Figure 10.**
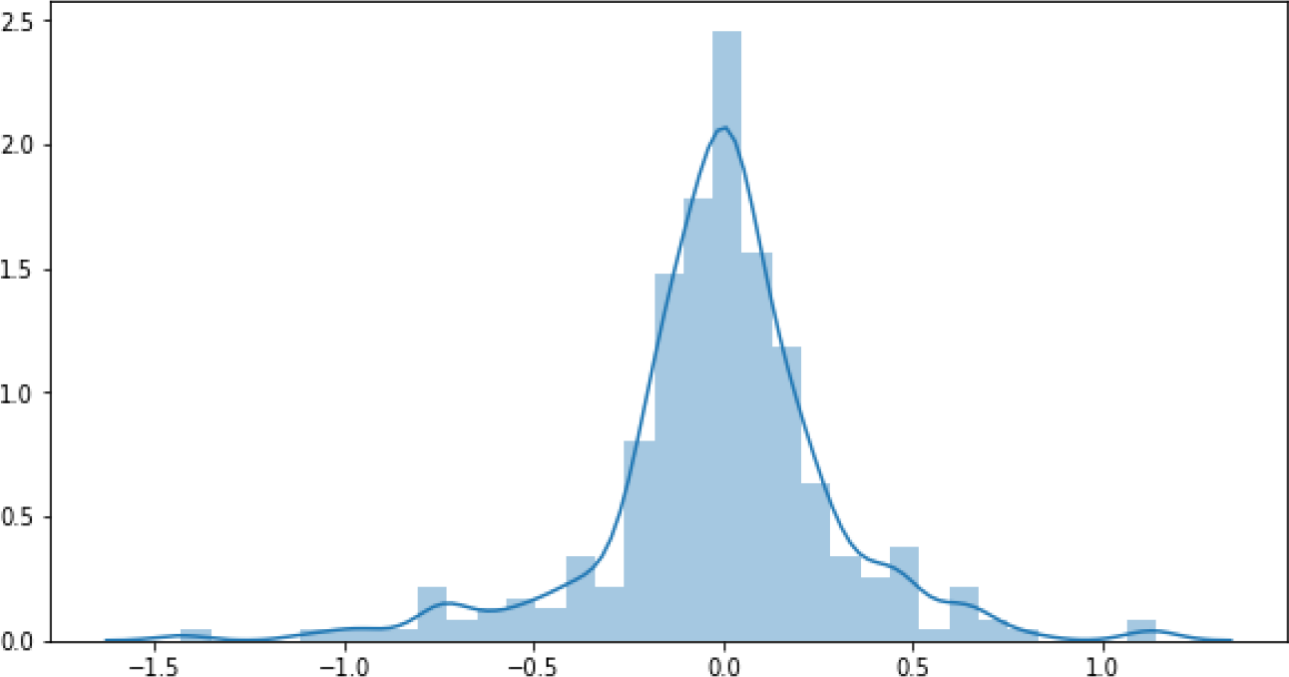
Optimised-DNNR residual.

Using the optimised-DNNR model on the dataset, a score of 0.91 was achieved with a total accuracy of around 94%. The MAE and the MSE rates are 0.20 and 0.09 respectively, which are in the acceptable loss range (error < 0.5). Figure 9 illustrates the residual plot of the optimised-DNNR model which shows a strong correlation between the predicted and the actual values. Figure 10 confirms that the optimised-DNNR model provides predictions within the loss range ±0.5.

## Discussion and conclusion

Recent popularity of mHealth and DNN enabled developing solutions to collect data from asthmatic patients and provide accurate predictive alerts. Although several studies support the association between weather and asthma, there is a lack of solutions for effective asthma self-management that can predict asthma exacerbation based on personalised weather triggers. This is due to three problems:

1. Limited availability of real-time weather data that can link weather triggers with demography and asthma severity for individual asthmatic patients. This study obtained the dataset from the WEA application which comprises relevant input features (weather and demography) and target output (asthma severity).
2. Existence of nonlinear relationships in the asthma dataset due to multiple types of input features and interconnected correlations. This study applied DNN for modelling the dataset, which effectively handles nonlinearity by using the ReLU activation function.
3. Lack of accurate predictive models and precautionary frameworks for effective asthma self-management. This study built an optimised model that provides accurate predictions of asthma exacerbation with errors in the acceptable loss range (error < 0.5).

The experimental results reveal that the standardisation technique improves the stability of the DNNR model, which enhances the performance of the optimisation algorithm and the optimiser. Furthermore, the fragmented-grid-search method is able to tune several parameters with much less computing time (≈26 minutes) than the standard grid-search used in previous studies (e.g. ≈4.3 hours for tuning 2 parameters ^22^). Moreover, model training takes less than one minute due to the Adam optimiser, which helps the model converge efficiently. Overall, the optimised-DNNR model provides predictions with a significantly higher accuracy rate (94%) than the existing ML models in the literature for predicting asthma exacerbation (e.g. 87% with naïve Bayes, ^2^ 85% through logistic regression, ^8^ and 84% using random forest ^23^).

Consequently, the optimisation process helps build an enhanced model for effective asthma self-management. Subsequently, the optimised model will be integrated into the WEA application for predicting asthma exacerbation based on personalised weather triggers and providing tailored feedback to users. The main limitation of this study is that the data was collected from a limited number of users and in one climate region. In future, more users from different climate regions will be considered for testing the generalisation capability of the proposed model.

### Author contribution

RH and SBH conducted the research, analysed the data, and wrote the paper. IC and AA improved and edited the paper. All authors have approved the final version.

## Data Availability

Zenodo. Dataset and source code for the research paper titled: “Optimised deep neural network model to predict asthma exacerbation based on personalised weather triggers”. DOI: https://doi.org/10.5281/zenodo.5271780. ^24^ Data are available under the terms of the Creative Commons Zero “No rights reserved” data waiver (CC BY 4.0 Public domain dedication).
