# Supplementary material for: Optimised deep neural network model to predict asthma exacerbation based on personalised weather triggers
Source: F1000Res. 2021 Sep 10;10:911. [Version 1] doi: 10.12688/f1000research.73026.1 (PMC8543171; doi:10.12688/f1000research.73026.1)
Supplement: Peer review history

# Peer review: Reviewer response for version 1

**DOI:** 10.5256/f1000research.76644.r95805

**Published:** 2021-11-02

**Authors:** Kwan-Yong Sim

**Affiliations:** 1 Swinburne University of Technology Sarawak Campus, Kuching, Malaysia

The main contribution of the paper is to identify the correlation patterns among asthma, weather, and demographic variables. Optimization of the DNNR model by applying standardization and fragmented grid search successfully reduced the error rate below 0.5 required for effective asthma self-management.

 Overall the paper is technically sound and made clear contributions to asthma self-management and improving the stability of the DNNR model proposed.

 The authors should include the following discussion to make the paper more cohesive:

1. As the study was conducted based on limited data collected from 10 users, the author should discuss the potential impact if a larger dataset is being used, including from different climate regions or different demographics.
2. "smoking habit" was mentioned as one of the features in data pre-processing. The author should explain what other features were included the data set (if any) and why they were included in the first place.

Is the work clearly and accurately presented and does it cite the current literature?

Yes

If applicable, is the statistical analysis and its interpretation appropriate?

Yes

Are all the source data underlying the results available to ensure full reproducibility?

Yes

Is the study design appropriate and is the work technically sound?

Yes

Are the conclusions drawn adequately supported by the results?

Yes

Are sufficient details of methods and analysis provided to allow replication by others?

Yes

Reviewer Expertise:

Data science, software testing,

I confirm that I have read this submission and believe that I have an appropriate level of expertise to confirm that it is of an acceptable scientific standard.

# Peer review: Reviewer response for version 1

**DOI:** 10.5256/f1000research.76644.r94416

**Published:** 2021-10-22

**Authors:** Meei Hao Hoo

**Affiliations:** 1 Department of Internet Engineering and Computer Science, University of Tunku Abdul Rahman, Kajang, Malaysia

The novelty of this study is to improve the accuracy of prediction of asthma by using optimised deep neural network and this gives a contribution to enhance the current model in terms of accuracy and computational performance. Most decisions in the research work are justifiable with supported of references made to the previous related works. Main results are explained in regards to the use of optimizer in building the model.

 In overall, the paper has a good-structured layout. However, the content in the method section can be improved to give a good flow of understanding. Either way to have an overall methodology of the study that describing the steps taken in the methods, or the arrangement and/or explanation in the methods follow the optimization process as in Figure 6.

 Suggest also to include relevant works to support the choice of ADAM optimizer in the study as compared with other optimizers, as well as the used of ReLU activation function over Mish (for example) and others in order to build strong arguments.

Is the work clearly and accurately presented and does it cite the current literature?

Partly

If applicable, is the statistical analysis and its interpretation appropriate?

Yes

Are all the source data underlying the results available to ensure full reproducibility?

Yes

Is the study design appropriate and is the work technically sound?

Yes

Are the conclusions drawn adequately supported by the results?

Yes

Are sufficient details of methods and analysis provided to allow replication by others?

Yes

Reviewer Expertise:

usability engineering, persuasive design and data mining.

I confirm that I have read this submission and believe that I have an appropriate level of expertise to confirm that it is of an acceptable scientific standard.

# Peer review: Reviewer response for version 1

**DOI:** 10.5256/f1000research.76644.r95806

**Published:** 2021-10-07

**Authors:** Mark Elshaw

**Affiliations:** 1 Computing. Electronics and Mathematics, Coventry University, Coventry, UK

This an interesting paper, which is timely. There is suitable use of deep learning, however there is a need to consider whether the problem is big data. It is good that there is some consideration of preprocessing. The paper follows a clear scientific process. There is a need to clearly outline why this paper is novel and to offer greater discussion of the future direction of the project.

 There is some indication of the performance achieved, but I would have liked to see greater statistical analysis. There is a need for consideration of the state of the art and how the approach in this paper compares with the state of the art.

 Overall a very well written and well presented paper that would benefit a little from greater analysis of the achievements. The analysis that you might consider using is the f-score, false positive and false negatives, and the use of ROC curves. **The developed approach would be of clear benefit to asthma patients.

Is the work clearly and accurately presented and does it cite the current literature?

Yes

If applicable, is the statistical analysis and its interpretation appropriate?

Partly

Are all the source data underlying the results available to ensure full reproducibility?

Yes

Is the study design appropriate and is the work technically sound?

Yes

Are the conclusions drawn adequately supported by the results?

Partly

Are sufficient details of methods and analysis provided to allow replication by others?

Partly

Reviewer Expertise:

Machine learning and robotics.

I confirm that I have read this submission and believe that I have an appropriate level of expertise to confirm that it is of an acceptable scientific standard.
